# First Chemical Profile Analysis of Acacia Pods

**DOI:** 10.3390/plants12193486

**Published:** 2023-10-05

**Authors:** Soraia I. Pedro, Tiago A. Fernandes, Ângelo Luís, Alexandra M. M. Antunes, José C. Gonçalves, Jorge Gominho, Eugenia Gallardo, Ofélia Anjos

**Affiliations:** 1 Polytechnic Institute of Castelo Branco, 6001-909 Castelo Branco, Portugal; soraia_p1@hotmail.com (S.I.P.); jcgoncalves@ipcb.pt (J.C.G.); 2 Centro de Biotecnologia de Plantas da Beira Interior, 6001-909 Castelo Branco, Portugal; 3 Centro de Química Estrutural (CQE), Institute of Molecular Sciences, Departamento de Engenharia Química, Instituto Superior Técnico (IST), Universidade de Lisboa, Avenida Rovisco Pais, 1049-001 Lisboa, Portugal; tiago.a.fernandes@tecnico.ulisboa.pt (T.A.F.); alexandra.antunes@tecnico.ulisboa.pt (A.M.M.A.); 4 Departamento de Ciências e Tecnologia (DCeT), Universidade Aberta,1000-013 Lisboa, Portugal; 5 Centro de Investigação em Ciências da Saúde (CICS-UBI), Universidade da Beira Interior, 6200-506 Covilhã, Portugal; afluis27@gmail.com (Â.L.); egallardo@fcsaude.ubi.pt (E.G.); 6 Laboratório de Fármaco-Toxicologia, UBIMedical, Universidade da Beira Interior, 6200-284 Covilhã, Portugal; 7 CERNAS-IPCB Research Centre for Natural Resources, Environment and Society, Polytechnic Institute of Castelo Branco, 6001-909 Castelo Branco, Portugal; 8 Centro de Estudos Florestais (CEF), Laboratório Associado TERRA, Instituto Superior de Agronomia, Universidade de Lisboa, 349-017 Lisboa, Portugal; jgominho@isa.ulisboa.pt

**Keywords:** *Acacia*, phenolic compounds, pods, UHPLC/ESI-QTOF-MS, HPLC-DAD, FT-Raman

## Abstract

This study intended to evaluate the potential industrial applications of various *Acacia* species (*Acacia melanoxylon*, *Acacia longifolia*, *Acacia cyclops*, *Acacia retinodes*, *Acacia pycnantha*, *Acacia mearnsii*, and *Acacia dealbata*) by examining their chemical composition, antioxidant, and antimicrobial properties. Using high-resolution mass spectrometry, a comprehensive analysis successfully identified targeted compounds, including flavonoids (flavonols/flavones) and phenolic acids, such as 4-hydroxybenzoic acid, *p*-coumaric acid, and ellagic acid. Additionally, *p*-coumaric acid was specifically identified and quantified within the hydroxycinnamic aldehydes. This comprehensive characterization provides valuable insights into the chemical profiles of the studied species. Among the studied species, *A. pycnantha* exhibited a higher concentration of total phenolic compounds, including catechin, myricetin, quercetin, and coniferaldehyde. Furthermore, *A. pycnantha* displayed notable antibacterial activity against *K. pneumoniae*, *E. coli*, *S. Typhimurium*, and *B. cereus*. The identified compounds in Acacia pods and their shown antibacterial activities exhibit promising potential for future applications. Moreover, vibrational spectroscopy was a reliable method for distinguishing between species. These significant findings enhance our understanding of *Acacia* species and their potential for various industrial applications.

## 1. Introduction

Many of the surrounding plants have been brought from their natural environment and are called exotic species. While a few of those plants co-exist in equilibrium with native species, others spread very quickly and beyond human control—invasive species. These can surpass geographical, abiotic, and biotic barriers, maintaining their invasive growth. *Acacia* spp. is one of those species that colonizes open spaces more quickly than native species, being then able to spread to other locations. This motivated the interest in valorizing the different parts of this invasive species to promote their harvest. Due to the potential of generated biomass, Da Costa et al. [1] focused their study on saccharification. The authors observed increased saccharification potential when mild alkali pretreatment was used and addressed the utilization of lignocellulose towards more sustainable production of biofuels and further biomaterials. Collected data adds value to underused biomass resources by characterizing biomass and pretreatments, hence contributing to establishing sustainable biorefining systems [1,2]. Some species, e.g., *Acacia mearnsii*, are capable of reducing the amount of methane produced by animals [3] and might be used as nutraceutical and preservatives to improve ruminant production and product quality, respectively [4] and potentially also usable in humans [5]. Their potential use as ingredients in cosmetics formulations [6] and for dyes production [7,8,9] has also been reported.

Additionally, some studies have explored their therapeutic potential [10], given that they are rich in secondary metabolites, including alkaloids, flavonoids, coumarins, saponins, and steroids [11]. Flavonoids are particularly interesting due to their potential as antioxidants, antibacterial, anti-thrombogenic, and antiviral agents [12]. Coherently, the antioxidant potential, enzyme inhibitory activities, and inhibition of microbial growth have been previously addressed [13,14,15,16]. Those studies have focused on using specific plant parts, such as wood, bark, seeds, leaves, flowers, and roots, with different contents and possible applications. However, scarce publications exist on the use and valorization of the plant’s pods. The purpose of this study was to assess the phenolic profile of different *Acacia* species’ pod extracts (*Acacia melanoxylon*, *Acacia longifolia*, *Acacia cyclops*, *Acacia retinodes*, *Acacia pycnantha*, *Acacia mearnsii*, and *Acacia dealbata*) to screen their different chemical composition and biological activities readily.

## 2. Results and Discussion

### 2.1. Determination of Total Phenolic and Flavonoid Contents

Phenolic compounds are considered the main secondary metabolites of plants, and they can be found in all plants [16]. These polyphenolic compounds are strong antioxidants responsible for various biological activities like antioxidant, anti-inflammatory, antidiabetic, antiaging, anticancer, and preventing cardiac diseases [16,17]. 

Table 1 shows the results for each species’ total phenolic content and flavonoids (dried weight). The results are expressed in milligrams of gallic acid equivalents per gram for the respective *Acacia* spp. extract.

The total phenolic content found in pods ranged from 0.12 (to *A. longifolia*) to 1.75 mg GAE/g extract (to *A. pycnantha*), and significant differences were found between the different species. Jelassi et al. [18] obtained higher values for TPC between 2.63 and 426.36 mg GAE/g DW. However, the authors used ethyl acetate as a solvent to obtain the extracts. 

Flavonoids have beneficial biological activities, specifically anti-inflammatory, antimicrobial antioxidant, cytotoxic, and antitumor [19]. This led us to determine the total flavonoid content (TFC) on the ethanolic extracts of the pods of all species included in this study by the aluminum chloride colorimetric method using a quercetin standard calibration curve (Table 1). The determined TFC ranged from 4.48 to 6.55 mg QE/g extract. Where maximum and minimum values were found for *A. longifolia* and *A. cyclops*, respectively.

The observed diversity in phenolic and flavonoid contents among plants can be attributed to various factors, including the influence of plant species, maturity stage, growing conditions, soil characteristics, and post-harvest treatments [20].

### 2.2. Determination of Antioxidant Properties

The antioxidant properties of the samples were evaluated using the DPPH free radical scavenging assay and the β-carotene bleaching test, and the results are presented in Table 2.

The DPPH radical scavenging assay has been commonly used to assess the antioxidant activity of plant extracts. In this study, the sample concentration necessary to decrease the initial concentration of DPPH by 50% (IC_50_) under the experimental condition was calculated. Thus, higher antioxidant activity is associated with samples with a lower value of IC_50_. The IC_50_ values of Acacia pods analyzed range from 1033 ± 7 to 8111 ± 571 mg/l, revealing a lower antioxidant activity.

In the β-carotene bleaching test, linoleic acid oxidation releases linoleic acid peroxide as free radicals that oxidize β-carotene, resulting in discoloration, thus decreasing the absorbance [21]. This method allows the indirect evaluation of the inhibition of lipid peroxidation by the extracts. The Acacia pod extracts showed to be poor antioxidants when compared to BHT. Our results contrast with previous studies suggesting that the green pods of *A. nilotica* are an important source of natural antioxidants [22].

### 2.3. Determination of Antimicrobial Activity

The antimicrobial activity of the extracts was evaluated against several strains of human pathogenic microorganisms. The disk diffusion assay measured the diameter of inhibition zones, presented in Table 3. Acacia pod extracts inhibited the growth of both Gram-positive and Gram-negative bacteria.

*K. pneumoniae* ATCC 13883 and *B. cereus* ATCC 11778 strains were the most susceptible to extract action, showing the biggest inhibition zones. This contrasts with what was observed for the Gram-positive strain of L. monocytogenes LMG 16779 and yeast, which were not susceptible to extract action. Nonetheless, these results suggest the potential antimicrobial application of these extracts.

After screening the antimicrobial properties of the extracts, their MIC values were determined by resazurin microtiter assay, and the results are presented in Table 4. The low MIC values obtained, particularly for *A. dealbata* and *A. pycnantha* extracts, attest to the antibacterial activity of the Acacia pod extracts against *K. pneumoniae* ATCC 13883 and *B. cereus* ATCC 11778.

### 2.4. LC-ESI-HRMS/MS Analysis 

LC-ESI-HRMS/MS was used for the targeted and unequivocal identification of specific flavonoids and flavones based on the similar retention time, the detection of the parent ion, and compatible tandem fragment ions when compared with their respective standards (Figures S1–S9).

**Figure 1:**
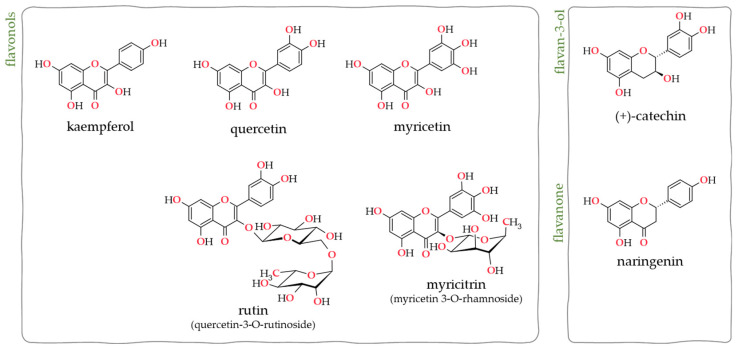
Structure of the seven flavonoid compounds identified in Acacia pods extracts LC-ESI-HRMS/MS analysis.

Seven flavonoids were identified: the flavonols myricetin, quercetin, kaempferol, rutin and myricitrin, the flavan-3-ol (+)-catechin, and the flavanone naringenin. ESI consistently detected all flavonoids in the positive and negative modes (Figure 1); however, flavonoids kaempferol, quercetin, and myricetin were only detected by ESI(+). Identifications were performed based on the identification of the parent adducted ions in the full scan spectra and on the recognition of suitable fragment ions in the tandem MS/MS spectra.

**Figure 2:**
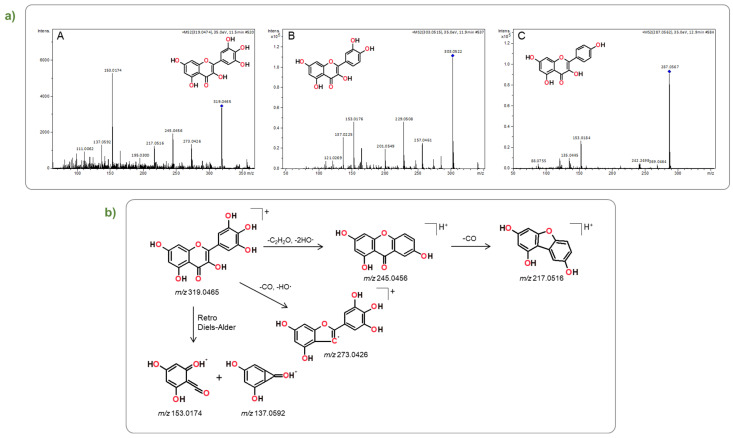
(**a**) Tandem Mass spectra, obtained by LC-ESI(+)-HRMS/MS, of flavonoids: (**A**) myricetin (*m*/*z* 319.0465); (**B**) quercetin (*m*/*z* 303.0522); and (**C**) kaempferol (*m*/*z* 287.0567); (**b**) Structures of predominant myricetin fragment ions, identified in the tandem H RMS spectra of this flavonol obtain by ESI(+).

Specifically, as expected, the protonated myricetin molecule was observed at *m*/*z* 319.0465 [23]. The identification of this flavonol was subsequently confirmed with a high degree of certainty by comparing its retention time and fragmentation patterns to those of a commercially available standard (Figure 2(aA) and Figure S3, Table 5 and Table S2) [24,25]. Figure 2b shows, for illustration purposes, the structures of the diagnostic fragment ions used for myricetin identification: the fragment ion at *m*/*z* 245.0456 undergoes a well-documented process [21], wherein three bonds within the C-ring are cleaved, leading to the formation of new bonds connecting the 6′-carbon of the B-ring and the 4-carbon of the C-ring The subsequent CO loss yields a new fragment ion at *m*/*z* 217.0516. At *m*/*z* 153.0174, is observed the characteristic MS/MS fragment, which stems from the retrocyclization on the A–C ring and the subsequent loss of CO. Quercetin identification was attested by the observation of its protonated molecule at *m*/*z* 303.0522, whose tandem mass spectrum exhibits the expected characteristic fragment ions (Figure 2(aB) and Figure S5, Table 5 and Table S2) [26]. Likewise, the tandem mass spectrum of the ion at *m*/*z* 287.0570, corresponding to the protonated molecule of kaempferol (C) (Figure 2(aC)), shows fragment ions compatible with the assigned structure: at *m*/*z* 269.0464, the ion formed following the loss of water from the parent protonated molecule; and at *m*/*z* 153.0184 the fragment ion stemming from a retro-Diels-Alder fragmentation [27] (Figure 2(aC) and Figure S5, Table 5 and Table S2).

**Figure 3:**
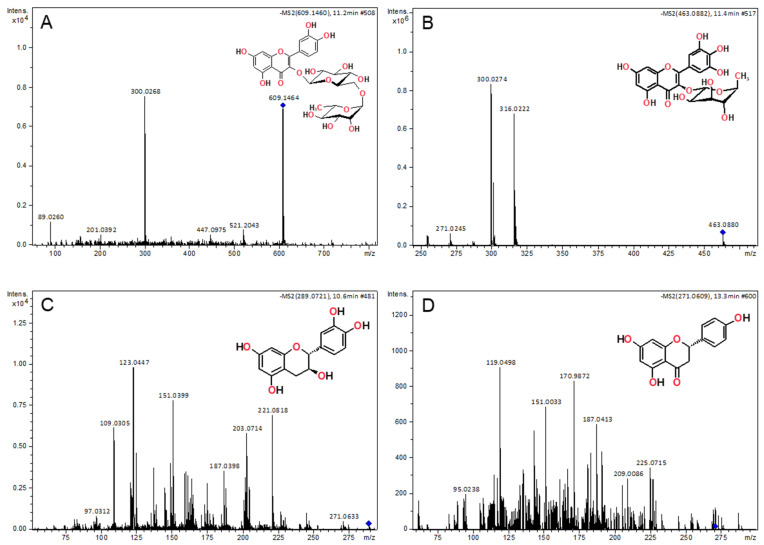
Tandem mass spectra of flavonoids (**A**) rutin (*m*/*z* 609); (**B**) myricitrin (*m*/*z* 463); (**C**) (+)-catechin (*m*/*z* 289) and (**D**) naringenin, obtained by LC-ESI(−)-HRMS/MS.

Rutin identification was attested by observing its deprotonated molecule at *m*/*z* 609.1464, [M−H]. Its MS/MS fragment ion at *m*/*z* 300.0263 corresponds to the characteristic loss of the glycoside moiety (Table 6) (Figure 3A) [28,29,30]. Moreover, the abundant myricetin-3-*O*-rhamnoside ion (at *m*/*z* 463.0880) was detected by ESI(−). Its fragmentation, formed by the homolytic cleavage of the O-rhamnosidic bond [M–H–C_6_H_11_O_4_]-(Figure 3B), results in the diagnostic fragment ion at *m*/*z* 316.0217, corresponding to the myricetin radical ion [31]. The deprotonated molecules of catechin and naringenin were observed at *m*/*z* 289.0721 and 271.0609, respectively, and their identification was confirmed upon standard comparison based on similar retention time and tandem mass spectra (Figure 3C,D).

We also searched for the presence of hydroxybenzoic acids, gallic acid, and 4-hydroxybenzoic acid. Both gallic acid and 4-hydroxybenzoic acid are examples of naturally occurring phenolic compounds. They are known to function in a wide variety of biological actions, such as antioxidant, anti-inflammatory, antibacterial, and anticancer properties [32]. 4-hydroxybenzoic acid identification was attested by observing its deprotonated molecule at *m*/*z* 137.0236, [M−H]^−^ and confirmed upon standard comparison. The observation of the benzenium fragment ion at *m*/*z* 93.0344, accompanied by the elimination of CHO_2_, is a distinctive feature specific to 4-hydroxybenzoic acid. This characteristic can differentiate it from similar compounds, including other phenolic acids or their derivatives [33] (Table 7). The presence of ellagic acid was also confirmed by standard comparison. The ellagic acid exhibited the [M−H]^−^ and [2M−H]^−^ ions with mass-to-charge ratios of 300.9993 and 602.9998, respectively. The tandem mass spectrum of the deprotonated molecule exhibited the expected fragmentation pattern, with peaks observed at *m*/*z* 283.9976 (indicating loss of a water molecule), 229.0144 (indicating loss of both CO_2_ and CO), and 185.0254 (indicating loss of two CO_2_ molecules and one CO molecule) [34]. The deprotonated molecule of *p*-coumaric acid was observed at *m*/*z* 163.0394, and it displays a fragment ion at *m*/*z* 119.0491 stems from the CO_2_ (44 u) neutral loss according to the α-elimination mechanism, which is a typical charge migration fragmentation in deprotonated compounds [35] (Table 7). The other fragment ion observed for *p*-coumaric at *m*/*z* 93.0351 is related to the acrylic acid (71 Da) neutral loss, which leads to the formation of the benzenium ion, similar to what was noticed for 4-hydroxybenzoic acid. Surprisingly, the *trans*-cinnamic acid was not found, which differs only by a hydroxyl group on the aromatic ring. Thus, during our meticulous search for target compounds, the presence of hydroxybenzoic acids (e.g., 4-hydroxybenzaldehyde, vanillin, syringaldehyde) and the simple phenol and catechols were also examined. However, there was no indication of their existence in the studied extracts that were examined as well.

### 2.5. HPLC-DAD Analysis

To quantify the compounds unequivocally identified by LC-HRMS/MS analysis, a simple methodology was used for simultaneously determining the compounds by a high-performance liquid chromatograph-diode (HPLC-DAD).

Table 8 summarizes the phenolic compounds identified and quantified for the different pod species, the wavelengths for each phenolic compound, and the retention times.

Table 8 reveals that *A. pycnantha* and *A. cyclops* exhibited the highest number of detected compounds among the species studied. In contrast, *A. retinodes* demonstrated the lowest number of observed compounds.

Rutin was found to be the compound with the highest concentration values for all species. However, it was *A. dealbata* that showed the highest concentration (25.91 µg/g). [17] also found similar results regarding this compound but for *A. nilotica*. When the concentrations of the compounds are compared, it can be concluded that *p*-coumaric acid and quercetin are found in all species, with a significantly higher concentration in *A. retinodes* and *A. dealbata*. Myricitrin concentrations were also observed to be higher in *A. retinodes*. 

To supplement the individual analysis based on the ANOVA results, an overall analysis was performed using cluster heat maps to visualize ranked clustering that ordered similar groups to understand the behavior of the various analyzed compounds concerning each species and discover consistent patterns among them. A heat map is a graphic representation of the data where the individual values in a matrix are represented as colors [16]. Since it is visually engaging and easy to read, this graphical representation has been used successfully to analyze relatively large data matrices.

**Figure 4:**
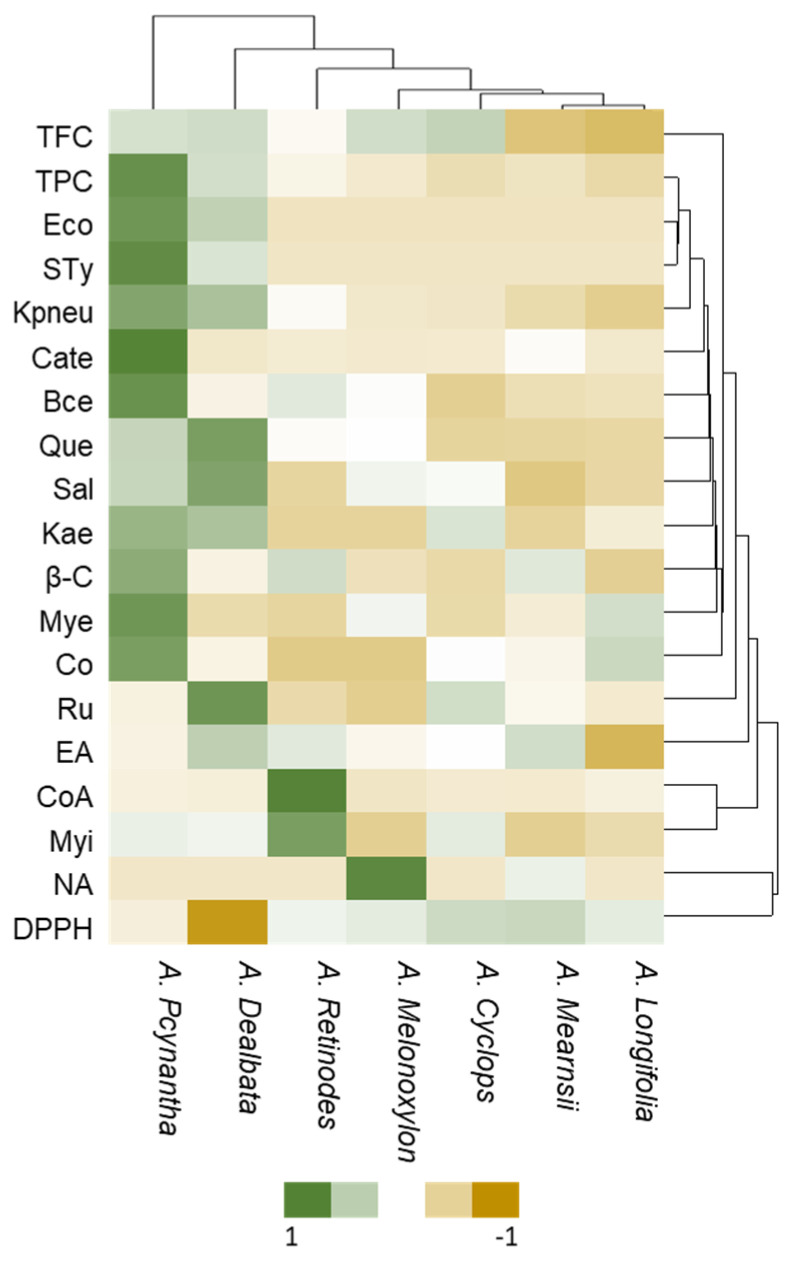
Heat maps plotting clusters of contents of phenol and flavonoid compounds, analyzed by HPLC of the *A. melanoxylon*, *A. longifolia*, *A. cyclops*, *A. retinodes*, *A. pycnantha*, *A. mearnsii*, *and A. dealbata* pods. Legend: TFC—total flavonoids compounds; TPC—total phenolic compounds; *β*-C—*β*-carotene; Cate—catechin; BA—4-Hydroxybenzoic acid; CoA—*p*-coumaric acid; Ru—rutin; Myi—myricitrin; EA—ellagic acid; Mye—myricetin; Que—quercetin; CO—coniferaldehyde; NA—naringenin; Kae -kaempherol; Eco—*E. coli*; Kpneu—*K. pneumoniae*; Sty—*S. Typhimurium*; Bce—*B. cereus*; Sal—*S. aureus*.

The heat map (Figure 4) was generated using the content of compounds quantified in each species, as well as the amount of phenol and flavonoid compounds and antibacterial properties. Green colors represent a positive correlation between analyte levels and species, while yellow colors represent a negative correlation.

Heat maps grouped the *Acacia* pod species into different clusters according to their chemical composition.

Total TPC, catechin, myricetin, quercetin, coniferaldehyde, and antibacterial activities against *E. coli*, S. Typhimurium, *K. pneumoniae*, and *B. cereus* are more prevalent in *A. pycnantha*, followed by *A. dealbata*, even though with some differences between them. For *A. dealbata*, there is a high correlation between quercetin and *S. aureus* and a weak positive link with *A. pcynantha*. The remaining compounds investigated demonstrate a modest negative connection. Some compounds are abundant in *A. retinodes* and *A. melanoxylon*; as previously stated, several are unique to this species. A notable example is naringenin, which has a weak negative correlation with all species except with *A. melanoxylon*. Another finding from the heat map analysis is that *A. mearnsii* and *A. longifolia* pods have similar chemical compositions and antibacterial profiles, and so do *A. melanoxylon* and *A. cyclops*.

### 2.6. FT-Raman Spectral Analysis 

**Figure 5:**
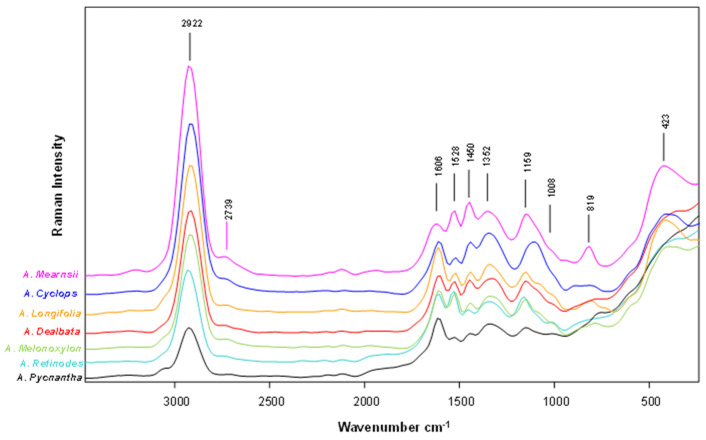
FT-Raman spectra of the lyophilized powder of *A. melanoxylon*, *A. longifolia*, *A. cyclops*, *A. retinodes*, *A. pycnantha*, *A. mearnsii*, and *A. dealbata* pods.

Raman spectroscopy is a vibrational technique helpful in identifying various compounds’ fingerprinting signatures. Figure 5 depicts the FT-Raman spectra obtained from the powder pods before extraction, displaying the most influenced bands for all *Acacia* species’ pods investigated. 

All spectra present a very representative and intense band at the 2922 cm^−1^ region. This band can be assigned to the stretch asymmetric C–H vibration of saturated hydrocarbons and hydrocarbon fragments [36,37,38,39]. Looking at the 1600 cm^−1^ region, all spectra reveal another important set of bands related to the aromatic ring C=C stretching vibrations at 1606 cm^−1^ and 1528 cm^−1^. These bands’ appearance depends on the position and nature of substituents on the aromatic ring [40]. Vibrational bands at 1606 cm^−1^ might originate from the lignin of plant cell walls, C=C linking, or by ν(C–C) aromatic ring [41]. The band at 1528 cm^−1^ might also have the contribution of N-H bend vibrations of primary and secondary amines, which are also observable in this region of the spectrum [36,37,38]. The Acacia green pods’ possible protein and lignin content might explain it.

When we examine the spectra at the lower wavelength zone, it is possible to identify many peaks ranging from 1550 cm^−1^ to 950 cm^−1^, yet all species exhibit some slight visible differences. The peak at 1450 cm^−1^ can be associated with the C–H bend and C=C–C aromatic ring stretch modes [38]. The band at 1352 cm^−1^ can be assigned to the C-N stretching band from the amides or amines [36,37,38]. This observed band can be due to the high protein content of acacia pods [42,43]. The peak at 1159 cm^−1^ could be attributed to the contribution of various vibrations, which can include C–O stretching bands from secondary alcohols, symmetric stretching vibration of carboxylic acid groups, C–C Stretching in glycosidic linkages, and C–O–C alkyl-substituted ether and esters [38,44]. The C–N stretching bands of aliphatic amines can be assigned to the weak shoulder at 1008 cm^−1^.

Nevertheless, this broadband can contribute to several vibration modes [37,39,45]. The signal at 819 cm^−1^ is usually linked to the aliphatic and aromatic C–H out-of-plane bend [37]. The peak at 423 cm^−1^ might be due to C–C–C and C–C–O deformations linked to glycosidic ring skeletal deformations τ(C–O) + δ(C–C–C) [46].

**Figure 6:**
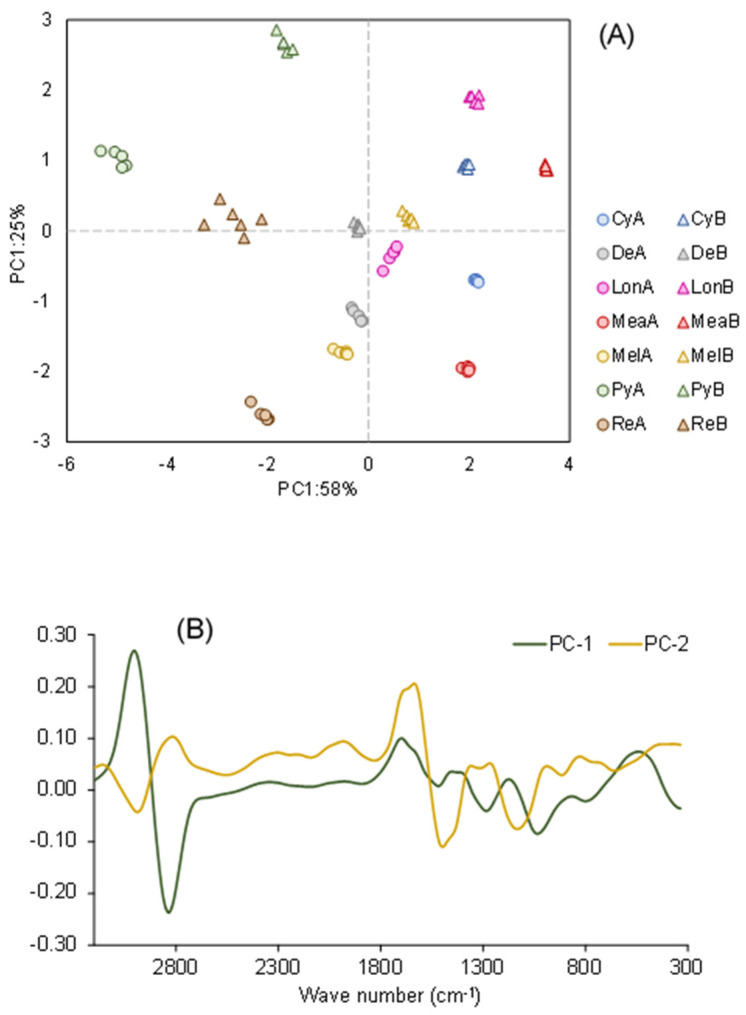
Score and lodgings plot of the first two principal components after the PCA performed with spectral information of the pod samples of different Acacias species studied, before (**A**) and after extraction (**B**) and corresponding loadings representation. Mel—*A. melanoxylon*; Lon—*A. longifolia*; Cy—*A. cyclops*; Re—*A. retinodes*; Py—*A. pycnantha*; Mea—*A. mearnsii*; De—*A. dealbata*.

Principal Component Analysis (PCA) was employed to discern the variations among the distinct species of Acacia pods through qualitative analysis (Figure 6). This PCA (mean-centered) is performed with the spectral information acquired with FT-Raman, using the algorithm’s first derivative of Savitzky-Golay and Singular value decomposition through a random cross-validation method with 20 segments.

The results plotted in Figure 6 show that the distinct acacia pods can be clearly distinguished from one another, as well as the pods following ethanolic extraction. The PCA obtained by the spectral analysis differs slightly from the differentiation made with the chemical and anti-microbiological analyses (Figure 1 and Table 4), indicating that more compounds must be found in the following studies. Therefore, FT-Raman spectroscopy revealed that it could be a valuable technique for monitoring the composition of acacia pods. However, more research will be needed for that. PCA from this data can recognize or discriminate between different raw materials.

## 3. Materials and Methods

### 3.1. Pods Material

The diverse distribution of the *Acacia* species in Portugal required the establishment of distinct geographic zones for gathering samples. The unripe green pods of *A. dealbata*, *A. melanoxylon, A. cyclops*, *A. retinodes*, *A. longifolia*, *A. pycnantha,* and *A. mearnsii* were collected in May 2021 in different regions of Portugal according to the scheme presented in [43]. After collecting the pods, they were left at room temperature until fully dry. Then, the material was immediately processed, and the samples were freeze-dried and kept at −80 °C until further use. In a hammer mill, the samples were reduced to a coarse powder (<2 mm).

### 3.2. Chemicals

Purified water was collected in a Milli-Qplus185 system (Millipore, Billerica, MA, USA). The reagents and standards (purity > 99.5%) obtained from the different suppliers are shown in Table S1.

Standard stock solutions in methanol (10 mg/L) were made and diluted with methanol to obtain working standard solutions. For LC-ESI-HRMS/MS, the standard solutions were newly prepared before use with ethanol/water (75:25 *v*/*v*), and all the solvents used (ethanol, water, and formic acid) for the chromatographic analysis were LC-MS grade and bought from Sigma-Aldrich (St. Louis, MO, USA).

### 3.3. Extraction Conditions

The freeze-dried sample (10 g) was extracted with 100 mL of ethanol (99%) on an orbital plate shaker for 24 h with constant stirring. After, all samples were: (1) filtrated; (2) centrifuged extract (4000× *g*, 20 min); (3) concentrated in a rotary evaporation system (with a temperature of 40 °C). The extractions were duplicated, and all the following analyses were performed in triplicate.

To determine the Minimum Inhibitory Concentration (MIC), the extracts were dissolved in a culture medium containing a maximum of 10% dimethyl sulfoxide (DMSO). Similarly, in the disc diffusion assay, the extracts were dissolved in dimethyl sulfoxide (DMSO).

### 3.4. Total Phenolic Compounds Determination

The Folin-Ciocalteu colorimetric technique was used to determine phenolics using a previously designed method [47]. Initially, each extract was diluted in methanol (50 µL), and then gallic acid (standard phenolic compound) was added and diluted with 450 µL of distilled water. After this procedure, 0.2 N Folin-Ciocalteu reagent (2.5 mL, diluted with distilled water) was added. 

The preparations were left to stand for 5 min, and Na_2_CO_3_ (2 mL, 75 g/L) aqueous solution was added. After incubation (90 min/30 °C), total phenolics were determined by colorimetry at 765 nm. A standard curve was performed using methanolic solutions of gallic acid with concentrations between 0.016 and 3.200 mg/L (y = 0.2249x; R^2^ = 0.9973). The total phenolic compound (TPC) content was expressed as mg gallic acid equivalents (GAE)/g extract. The tests were performed in triplicate.

### 3.5. Flavonoid Contents Determination 

To estimate flavonoid content, the aluminum chloride colorimetric method was used according to a protocol in the literature [47].

Each extract solution (500 μL) was mixed with 1.5 mL of methanol, 0.1 mL of 10% (*w*/*v*) aluminum chloride, 0.1 mL of potassium acetate (1 M), and 2.8 mL of distilled water. In this method, quercetin was used as a standard.

The solutions were allowed to stand for 30 min at room temperature. Then, using a spectrophotometer, the absorbance of the reaction mixture was measured at 415 nm. A standard curve was developed with methanolic solutions by preparing eight quercetin solutions with concentrations ranging from 2.5 to 12.5 mg/L (y = 0.0557x; R^2^ = 0.9925). The quantification of total flavonoid content was conducted by expressing the values as milligrams of quercetin equivalents per gram of extract (mg QE/g extract). The determinations mentioned above were performed in triplicate.

### 3.6. Evaluation of Antioxidant Properties

The β-carotene/linoleic acid system and DPPH method evaluated the antioxidant activity using butylated hydroxytoluene (BHT) or gallic acid as standards, respectively.

#### 3.6.1. DPPH Scavenging Assay

The extracts’ antioxidant activity was determined using the 2,2-Diphenyl-1-picrylhydrazyl (DPPH) free radical scavenging activity method [47]. Extracts or standards (0.1 mL) at different concentrations were added to a methanolic solution of DPPH (3.9 mL). Three solutions with a concentration of 0.2000, 0.1242, and 0.0800 mM of DPPH were tested. These solutions were prepared by dissolving 39.4, 24.5, and 15.8 mg in 500 mL of DPPH in methanol. The control sample consisted of a 0.1 mL solution of methanol stirred with 3.9 mL of DPPH. Subsequently, the absorbance was measured at 517 nm after a 90-min incubation period at room temperature in the dark. 

The radical scavenging activity was calculated using the formula: I% = [(Abs_0_ − Abs_1_)/Abs_0_] × 100. In this equation, Abs_0_ represents the absorbance value of the control, while Abs_1_ represents the absorbance value obtained when the test sample was present at various concentrations.

The IC_50_ was calculated graphically using a linear calibration curve, plotting the concentration of the test sample at different concentrations. Linear plotting the concentration of the extract vs. the corresponding knockout effect. 

The quantification of antioxidant activity is expressed by the Antioxidant Activity Index (AAI), which was determined through the following calculation: AAI = (final concentration of the final DPPH concentration in the control sample)/(IC_50_) [47]. Various levels of antioxidant activity were taken into consideration. The activity level can be categorized as weak when the AAI (Activity Index) is less than 0.5. Moderate activity is observed when the AAI falls between 0.5 and 1.0. Strong activity is indicated when the AAI ranges from 1.0 to 2.0. Lastly, very strong activity is identified when the AAI exceeds 2.0 [47]. All tests were conducted in triplicate.

#### 3.6.2. β-Carotene Bleaching Test

After preparation, a volume of 500 µL of a *β*-carotene solution (500 mg/mL in chloroform) was added to 40 µL of linoleic acid, 400 µL of Tween 40, and 1 mL of chloroform. This mixture was evaporated at 45 °C in rotary evaporation for 5 min to remove chloroform and diluted with oxygenated distilled water (100 mL). Then, (5 mL) were pipetted into test tubes containing the extracts at different concentrations (300 μL) of the previous emulsion. The control consisted of 5 mL of the emulsion, 300 µL of methanol, and standard butylated hydroxytoluene (BHT) in methanol at the same concentration as samples and was used as a reference. Finally, the tubes were shaken and placed at 50 °C in a water bath for 1 h. The samples’ absorbances were measured at 470 nm, using a spectrophotometer, against a blank consisting of an emulsion without β-carotene. The measurements were carried out at the initial time (t = 0 h) and at the final time (t = 1 h). The antioxidant activity was measured in terms of the percentage of inhibition of β-carotene’s oxidation by % Inhibition = (Abs^t=1^ _sample_ − Abs^t=1^ _control_)/(Abs^t=0^ _control_ − Abs^t=1^ _control_). Where Abs^t=1^ was the absorbance of the sample or control at the final time of incubation, and Abs^t=0^ was the absorbance in control at the initial time of incubation [47].

### 3.7. Determination of Antimicrobial Activity

#### 3.7.1. Test Microorganisms and Culture Media

The antimicrobial activity of the extracts was evaluated against several microbial strains: Gram-negative bacteria (*Klebsiella pneumoniae* ATCC 13883, *Salmonella* Typhimurium ATCC 13311, *Escherichia coli* ATCC 2592), Gram-positive bacteria (*Staphylococcus aureus* ATCC 25923, *Bacillus cereus* ATCC 11778, *Listeria monocytogenes* LMG 16779) and one yeast (*Candida albicans* ATCC 90028). The microbial strains’ stock cultures were maintained in 20% glycerol at −80 °C. The yeast strain was sub-cultured in Sabouraud Dextrose Agar (SDA) and the bacterial strains in Brain-Heart Infusion agar (BHI) 24 h before the antimicrobial testing.

#### 3.7.2. Disc Diffusion Assay

The disc diffusion method was employed to evaluate the antimicrobial activity of the extracts following the Clinical Laboratory and Standards Institute (CLSI) standard protocols (M2-A8 for bacteria and M44-A2 for yeasts). The inoculums were prepared by suspending several microbial colonies in a sterile saline solution, which adjusted turbidity to 0.5 McFarland. Sterile cellulose discs (6 mm diameter) were saturated with 20 μL of the extracts dissolved in DMSO at 200 mg/mL (4 mg/disc), which were then placed on the inoculated agar plates. DMSO (20 μL/disc) was used as a negative control, and tetracycline (30 µg/disc) or amphotericin B (25 µg/disc) were used as positive controls. The bacterial plates were incubated for 24 h at 37 °C, and those inoculated with the yeast were incubated for 48 h at 37 °C. Using a digital pachymeter, the inhibition zone diameters were measured millimeters after the incubation period. This assay was conducted three independent times [47].

#### 3.7.3. Resazurin Microtiter Method

The resazurin microtiter method was employed to determine the extracts’ Minimum Inhibitory Concentration (MIC). Initially, for bacterial strains, 100 µL of each extract (20 mg/mL in Müeller-Hinton Broth (MHB), and 10%, *v*/*v*, DMSO) was added to the first row of a 96-multiwell plate. Then, 50 µL of MHB was added to the other wells. Following, serial two-fold dilutions were completed with a multichannel pipette, the tips discarded with the last 50 µL; 10 µL of the resazurin indicator solution (0.1%, *w*/*v*, MHB) and 30 µL of MHB were added to all the plate wells. Ultimately, 10 µL of the inoculums ascertained at 0.5 McFarland were also added to the wells. Several controls were considered: an antibiotic used a positive control, a column with all solutions except the extracts, and a column with all solutions except the inoculums. The plates were incubated for 18 h at 37 °C and were prepared in independent triplicates. 

In the case of the yeast, the working suspension (inoculum diluted 1:1000 in culture medium) was supplemented with resazurin solution (50 µL, 20 mg/mL in water). The culture medium used for yeasts was RPMI-1640, supplemented with glutamine and phenol red, without bicarbonate, and buffered with 3-(*N*-morpholino)propane sulfonic acid (MOPS). Microdilution susceptibility testing was also conducted, changing the final volume in the wells to 200 µL.

Positive observations were made when the color transitioned from purple to pink or became colorless. The minimum inhibitory concentration (MIC) value was determined as the concentration at which the first observable change occurred [47].

### 3.8. Chemical Analysis

#### 3.8.1. LC-ESI-HRMS/MS Analysis

Representative phenolic compounds were subsequently identified by a previously developed methodology using LC-ESI-HRMS/MS [34]. The ethanolic extracts were subjected to analysis using an Elute UPLC system (Bruker, Bremen, Germany) that was connected to a Bruker Impact II quadrupole time-of-flight mass spectrometer equipped with an electrospray ionization (ESI) source (Bruker Daltoniks, Bremen, Germany). A Luna C18 column (3.0 µm, 2.0 × 150 mm; Phenomenex) was used for the chromatographic separation. A flow rate of 170 μL/min was employed in the experiment, with the mobile phase comprising two components: 0.1% formic acid in water (referred to as mobile phase A) and 0.1% formic acid in acetonitrile (referred to as mobile phase B). The elution program followed a specific sequence: 5–50% B for a period of 6 min, 50–100% B for 4 min, isocratic elution with 100% B for 5 min, 100–5% B for 4 min, and finally 5% B for 9 min. The WS samples went through filtration without any supplementary preparation by employing a sterile syringe filter with a hydrophilic PVDF membrane of 0.45 µm pore size and 25 mm diameter before analysis. The volume of the injection was 10 μL. The autosampler and column were maintained at 8 °C and 40 °C, respectively. Spectra were acquired in the positive ESI(+) and negative ESI(−) electrospray ionization modes. The following mass spectrometric parameters were used: end plate offset, 500 V; capillary voltage, (±)4.5 kV; nebulizer, 40 psi; dry nitrogen gas, 8 L/min; heater temperature, 200 °C. The sodium formate cluster was used for internal calibration using high-precision calibration mode (HPC). The acquisition was conducted in the *m*/*z* 50–1000 range by data-dependent MS/MS mode with a 0.5 isolation window, a 3 Hz acquisition rate, and a fixed cycle length of 3 s. At an absolute threshold of 153, precursor ions were chosen for auto MS/MS, with the active exclusion mode set to three spectra and released after 1 min. However, precursor ions with intensities up to five times higher than previous intensities were examined.

#### 3.8.2. HPLC-DAD Analysis

A previously validated methodology [16] was used to quantify the predominant phenolic compounds in the extracts through HPLC-DAD. Standard solutions of each phenolic compound (4-hydroxybenzoic acid, ellagic acid, *p*-Coumaric acid, coniferaldehyde, (+)-catechin, rutin, myricitrin, myricetin, quercetin, kaempferol and naringenin) were diluted with ethanol to the final concentration of 1 mg/mL and stored at 4 °C in the dark.

For sample analysis, a high-performance liquid chromatography system with a diode array detector (HPLC-DAD) by Agilent Technologies was employed. The dried samples were dissolved in 500 µL of ethanol and filtered through a 0.22 µm filter before being injected into the chromatographic system. Chromatographic separation occurred on a YMC-Triart PFP (150 × 4.6 mm, 5 µm i.d.) with pre-column (Solitica, Arruda dos Vinhos, Portugal). 

The mobile phase consisted of acetonitrile (A) and 0.1% trifluoroacetic acid in water (B)in a gradient mode: 10% A (0–3 min), 10- 15% A (3–15 min), 15% A (15–20 min), 15–18% A (20–25 min), 18–30% A (25–40 min), 30–50% A (40–45 min), 50–100% A (45–50 min); and returning to, 10% A (50–55 min). The flow rate was 1 mL/min, with a 50 μL injection volume. The column and sampler temperatures were 35 and 4 °C, respectively. Detected analytes have absorbance within the 255 to 360 nm wavelength range.

For the isolation of rutin and myricitrin, the mobile phase comprised (A) 0% acetonitrile and (B) 100% orthophosphoric acid, using a gradient mode: 0% A (0–2 min), 9% A (2–14 min), 13% A (14–22 min), 33% A (22–38 min), and 43% A (38–44 min), maintained 43% (44–55) min, and returning to 0% A (55–65 min). The flow rate was 0.8 mL/min, with a 50 µL injection volume. The column and sampler temperatures were set at 24 °C and 4 °C, respectively. Rutin and myricitrin were detected at 255 and 263 nm, respectively. 

The identification of the target compounds was accomplished by comparing their retention times with those obtained from analytical standards. The identified compounds were quantified by comparing their peak areas observed in the extract chromatograms with calibration curves constructed using the respective standard solutions.

#### 3.8.3. Vibrational Spectroscopy

The methodology described in a previous study, [48] was employed to obtain the spectra of the extracts from the pods. An FT-Raman spectrometer (BRUKER, MultiRAM, Bruker Portugal Unipessoal, Lisbon, Portugal) equipped with a 180 high-throughput collecting lens, an ultra-high sensitivity liquid nitrogen cooled Ge Diode detector, and an integrated 1064 nm diode-pumped Nd: YAG laser with a maximum output power of 500 mW was utilized for this purpose. The spectra acquisition was conducted under the following conditions: 64 scans per spectrum at a spectral resolution of 32 cm^−1^, scanner velocity of 5 kHz, and wavenumber range from 4000 to 200 cm^−1^. Spectra were acquired on the powder for each species before and after extraction. The measurements were duplicated in an 8 mm optic space quartz cell with the opposite face mirrored.

### 3.9. Data Analysis

The statistical technique employed in this study was a one-way analysis of variance (ANOVA) to assess the presence of any statistically significant disparities among the measured parameters in the various samples. The least significant difference (LSD) was used in the context of the ANOVA to determine that each individual means differed from the others. STATISTICA 7 software (StatSoft Inc., Tulsa, OK, USA) was used to perform the analysis above.

For spectral data analysis, OPUS^®^, version 7.5.18 (Bruker Optics, Ettlingen, Germany) and UnscramblerX 10.5 (CAMO, Oslo, Norway) were used.

The acquired data by LC-ESI-HRMS/MS were processed by Data Analysis 4.1 software (Bruker Daltoniks). Extracted ion chromatograms (EIC) were conducted with a mass window of ±5 ppm to identify characteristic fragment ions associated with 4-hydroxybenzoic acid, ellagic acid, *p*-coumaric acid, (+)-catechin, rutin, myricitrin (myricetin 3-*O*-rhamnoside), myricetin, quercetin, kaempferol, and naringenin in the tandem mass spectra. The discrepancy in mass between the identified metabolites and their accurate mass was consistently within a range of less than 5 ppm for precursor ions and less than 10 ppm for product ions. In the Supplementary Material, the MS/MS spectra of the products identified are displayed.

## 4. Conclusions

With the ultimate aim of assessing the suitability of Acacia species for industrial applications, this study successfully examined the phenolic composition, as well as the antioxidant and antimicrobial properties of extracts obtained from seven Acacia species. Using LC-ESI-HRMS/MS, even compounds belonging to different organic families were unequivocally identified and subsequently quantified using HPLC-DAD. Flavonoids (flavonols/flavones) and phenolic acids contents, namely *p*-coumaric acid, 4-hydroxybenzoic acid, and ellagic acid, were employed to differentiate between the various species of *Acacia* pods under investigation. Even though there are some differences between each analyzed *Acacia* spp., *A. pcynantha* has more total phenolic compounds, catechin, myricetin, quercetin, coniferaldehyde, and antibacterial activities against *E. coli*, *K. pneumoniae*, *S.* Typhimurium, and *B. cereus*. Less undoubtedly, these more prevalent results were also found in *A. dealbata*. The total phenolic content of pods ranged from 0.12 to 1.75 mg GAE/g extract, with considerable variances between species. Whereas *A. pycnantha* had the highest concentration and *A. longifolia* had the lowest. The total flavonoid content varied from 4.48 to 6.55 mg QE/g extract, with the maximum value obtained for *A. longifolia* and the lowest value for *A. cyclops*. The low MIC values obtained, notably for *A. dealbata* and *A. pycnantha* extracts, demonstrate the *Acacia* pods extracts’ antibacterial efficacy against *K. pneumoniae* ATCC 13883 and *B. cereus* ATCC 11778. The compounds identified in *Acacia* pods show potential for future applications, and vibrational spectroscopy was found to be a reliable method for distinguishing between species. These findings contribute to understanding *Acacia* species and their potential industrial uses.

## Figures and Tables

**Table 1:** Total phenolic compounds and flavonoid content of the seven *Acacia* species pods extracts (mean ± standard deviation).

*Acacia* species	TPC (mg GAE/g Extract)	TFC (mg QE/g Extract)
*A. melanoxylon*	0.32 ± 0.02 ^c^	6.38 ± 0.42 ^b^
*A. longifolia*	0.12 ± 0.00 ^a^	4.48 ± 0.13 ^a^
*A. cyclops*	0.17 ± 0.00 ^ab^	6.55 ± 0.15 ^b^
*A. retinodes*	0.44 ± 0.02 ^d^	5.66 ± 0.17 ^ab^
*A. pycnantha*	1.75 ± 0.06 ^f^	6.32 ± 0.58 ^b^
*A. mearnsii*	0.25 ± 0.06 ^bc^	4.62 ± 0.86 ^a^
*A. dealbata*	0.92 ± 0.01 ^e^	6.39 ± 0.22 ^b^

TPC—otal phenolic compounds; TFC—Total flavonoid content; GAE—gallic acid equivalents; QE—quercetin equivalents. Means within the same column followed by different letters are significantly different (*p* < 0.05) according to the LSD Test.

**Table 2:** Antioxidant properties of the seven Acacia species pod extracts were measured using two methods (mean ± standard deviation).

*Acacia* Species	DPPH Free Radical Scavenging Assay		*β*-Carotene Bleaching Test
	IC_50_ (mg/L)	AAI	IC_50_ (mg/L)
*A. melanoxylon*	7165 ± 730 ^bc^	0.00 ± 0.00 ^a^	3526 ± 1997 ^abc^
*A. longifolia*	7158 ± 741 ^bc^	0.00 ± 0.00 ^a^	6582± 547 ^cd^
*A. cyclops*	7969 ± 474 ^c^	0.01 ± 0.00 ^a^	6048 ± 961 ^bcd^
*A. retinodes*	6815 ± 516 ^bc^	0.01 ± 0.00 ^a^	2890 ± 395 ^a^
*A. pycnantha*	5399 ± 2519 ^b^	0.04 ± 0.05 ^a^	4397 ± 398 ^abc^
*A. mearnsii*	8111 ± 570 ^c^	0.01 ± 0.00 ^a^	8786 ± 2817 ^d^
*A. dealbata*	1033 ± 7 ^a^	0.04 ± 0.00 ^a^	3148 ± 208 ^ab^
Positive control—BHT	-	-	78.0 ± 6.2

IC_50_—half maximal inhibitory concentration; AAI—antioxidant activity index; BHT—butylated hydroxytoluene; DPPH—2,2-diphenyl-1-picrylhydrazyl. Data expressed as means ± standard deviation (SD) of triplicate assays; Mean values in a column with different letters are significantly different (*p* < 0.05).

**Table 3:** Diameter of inhibition zones (mm) of Acacia pod extracts.

*Acacia* Species (4 mg/Disk)	Gram-Negative Bacteria			Gram-Positive Bacteria			*Candida albicans* ATCC 90028
	*Escherichia coli* ATCC 25922	*Klebsiella pneumoniae* ATCC 13883	*Salmonella* Typhimurium ATCC 13311	*Bacillus cereus* ATCC 11778	*Staphylococcus aureus* ATCC 29213	*Listeria monocytogenes* LMG 16779	
*A. melanoxylon*	6.00 ± 0.00 ^a^	9.26 ± 0.41 ^c^	6.00 ± 0.00 ^a^	9.19 ± 0.26 ^c^	10.59 ± 0.78 ^b^	6.00 ± 0.00	6.00 ± 0.00
*A. longifolia*	6.00 ± 0.00 ^a^	7.98 ± 0.36 ^a^	6.00 ± 0.00 ^a^	8.07 ± 0.43 ^b^	8.12 ± 0.12 ^a^	6.00 ± 0.00	6.00 ± 0.00
*A. cyclops*	6.00 ± 0.00 ^a^	9.25 ± 0.35 ^c^	6.00 ± 0.00 ^a^	7.17 ± 0.19 ^a^	10.27 ± 0.61 ^b^	6.00 ± 0.00	6.00 ± 0.00
*A. retinodes*	6.00 ± 0.00 ^a^	10.55 ± 0.20 ^d^	6.00 ± 0.00 ^a^	9.94 ± 0.41 ^c^	8.14 ± 0.74 ^a^	6.00 ± 0.00	6.00 ± 0.00
*A. pycnantha*	8.26 ± 0.29 ^c^	14.47 ± 0.47 ^f^	9.36 ± 0.06 ^c^	12.76 ± 0.23 ^d^	12.07 ± 0.19 ^c^	6.00 ± 0.00	6.00 ± 0.00
*A. mearnsii*	6.00 ± 0.00 ^a^	8.52 ± 0.52 ^b^	6.00 ± 0.00 ^a^	8.27 ± 0.91 ^b^	7.35 ± 0.18 ^a^	6.00 ± 0.00	6.00 ± 0.00
*A. dealbata*	7.56 ± 0.51 ^b^	14.39 ± 0.61 ^e^	7.30 ± 0.06 ^b^	8.41 ± 0.36 ^b^	14.20 ± 0.44 ^d^	6.00 ± 0.00	6.00 ± 0.00
DMSO (20 µL/disk)	6.00 ± 0.00	6.00 ± 0.00	6.00 ± 0.00	6.00 ± 0.00	6.00 ± 0.00	6.00 ± 0.00	6.00 ± 0.00
Tetracycline (30 µg/disk)	23.25 ± 0.50	22.25 ± 0.50	11.50 ± 0.58	30.00 ± 0.82	30.25 ± 0.50	18.25 ± 0.60	-
Amphotericin B (25 µg/disk)	-	-	-	-	-	-	20.33 ± 0.58

Data is presented as means ± standard deviation (SD) of triplicate assays. Mean values in a column that are denoted by different letters are found to be significantly different at a significance level of (*p* < 0.05).

**Table 4:** MIC values (mg/mL) of Acacia pods extracts.

*Acacia* Species	Gram-Negative Bacteria			Gram-Positive Bacteria			*Candida albicans* ATCC 90028
	*Escherichia coli* ATCC 25922	*Klebsiella pneumoniae* ATCC 13883	*Salmonella* Typhimurium ATCC 13311	*Bacillus cereus* ATCC 11778	*Staphylococcus aureus* ATCC 29213	*Listeria monocytogenes* LMG 16779	
*A. melanoxylon*	>10	10	>10	1.25	10	>10	>10
*A. longifolia*	>10	>10	>10	5	10	>10	>10
*A. cyclops*	>10	10	>10	2.5	>10	>10	>10
*A. retinodes*	>10	10	>10	1.25	>10	>10	>10
*A. pycnantha*	5	2.5	10	0.313	2.5	>10	>10
*A. mearnsii*	>10	>10	>10	1.25	10	>10	>10
*A. dealbata*	5	2.5	10	0.625	5	>10	>10
Tetracycline (µg/mL)	0.06	0.06	0.24	0.06	0.06	0.06	-
Amphotericin B (µg/mL)	-	-	-	-	-	-	0.25

Modal values of triplicate assays.

**Table 5:** Flavonols identified by LC-ESI(+)-HRMS/MS.

Identified Compound	RT^a^ (min)	Molecular Formula	[M+H]^+^ (*m*/*z* Exp)	Error (ppm)	Fragment Ions *m*/*z* (Error ppm, Molecular Formula)
kaempferol	13.2	C_15_H_10_O_6_	287.0567	5.9	269.0464 (7.2, C_15_H_9_O_5_^+^) 153.0184 (1.1, C_7_H_5_O_4_^+^) 135.0445 (3.3, C_8_H_7_O_2_^+^)
quercetin	13.0	C_15_H_10_O_7_	303.0522	7.5	257.0461 (6.4, C_14_H_9_O_5_^+^) 229.0508 (5.5, C_13_H_9_O_4_^+^) 201.0549 (1.4, C_12_H_9_O_3_^..+^) 153.0176 (−4.1, C_7_H_5_O_4_^+^) 137.0225 (−6.0, C_7_H_5_O_3_^+^)
myricetin	11.5	C_15_H_10_O_8_	319.0465	5.2	273.0426 (11.8, C_14_H_9_O_6_^+^) 245.0456 (0.4, C_13_H_9_O_5_^+^) 217.0516 (9.5, C_12_H_9_O_4_^+^) 153.0174 (−5.5, C_7_H_5_O_4_^+^) 137.0592 (−3.7, C_8_H_9_O_2_^+^)

**Table 6:** Targeted identification of flavonoid compounds by LC-ESI(−)-HRMS/MS.

Identified Compound	RT^a^ (min)	Molecular Formula	[M−H]^−^ (*m*/*z* Exp)	Error (ppm)	Fragment Ions, *m*/*z* (Error ppm, Molecular Formula)
rutin	11.2	C_27_H_30_O_16_	609.1464	0.5	300.0263 (−4.2, C_15_H_8_O_7_^●−^)
myricitrin	11.4	C_21_H_20_O_12_	463.0880	−0.4	316.0222 (−0.8, C_15_H_8_O_7_^●−^) 300.0263 (−4.2, C_15_H_8_O_7_^●−^)
(+)-catechin	10.6	C_15_H_14_O_6_	289.0721	1.2	245.0828 (3.5, C_14_H_13_O_4_^−^) 221.0818 (−0.6, C_12_H_13_O_4_^−^) 203.0714 (0.2, C_12_H_11_O_3_^−^) 187.0398 (−1.4, C_11_H_7_O_3_^−^) 151.0399 (2.0, C_7_H_3_O_4_^−^) 123.0447 (−3.6, C_7_H_7_O_2_^−^) 109.0305 (9.1, C_6_H_5_O_2_^−^)
naringenin	13.4	C_15_H_12_O_5_	271.0609	−1.1	187.0413 (6.5, C_11_H_7_O_3_^−^) 119.0498 (−3.7, C_8_H_7_O^−^) 151.0033 (−2.5, C_7_H_3_O_4_^−^)

**Table 7:** Targeted identification of phenolic compounds by LC-ESI(−)-HRMS/MS.

Identified Compound	RT^a^ (min)	Molecular Formula	[M−H]^−^ (*m*/*z* Exp)	Error (ppm)	Fragment Ions *m*/*z* (Error ppm, Molecular Formula)
4-hydroxybenzoic acid	13.4	C_7_H_6_O_3_	137.0236	−6.0	93.0344 (−2.0, C_6_H_5_O^−^)
ellagic acid	11.7	C_14_H_6_O_8_	300.9989 602.9995 [2M−H]^−^	−0.3	283.9976 (4.7, C_14_H_4_O_7_^−^) 229.0144 (0.7, C_12_H_5_O_5_^−^) 185.0254 (5.3, C_11_H_5_O_3_^−^)
*p*-coumaric acid	12.0	C_9_H_8_O_3_	163.0394	−4.1	191.0491 (−5.0, C_8_H_7_O^−^) 93.0351 (5.5, C_6_H_5_O^−^)

**Table 8:** The concentration of phenolic compounds (µg/g) in the ethanol extracts from the seven *Acacia* species pods by HPLC-DAD using analytical standards and calibration curves (mean ± standard deviation).

Compound	t_R_ (min)	λ_max_ (nm)	De	Lon	Re	Py	Mel	Cy	Mea
Hydroxybenzoic acids									
4-hydroxybenzoic acid	9.8	255	<LOQ ▪	<LOQ ▪	<LOQ ▪	<LOQ ▪	0.21 ± 0.03 ^b^	<LOQ ▪	0.06 ± 0.00 ^a^
ellagic acid	25.66	255	0.57 ± 0.53 ^a^	<LOQ ▪▪	0.45 ± 0.00 ^a^	0.30 ± 0.00 ^a^	0.32 ± 0.00 ^a^	0.35 ± 0.06 ^a^	<LOQ ▪▪
Hydroxycinnamic acids									
*p*-coumaric acid	19.8	291	0.40 ± 0.04 ^a^	0.47 ± 0.03 ^a^	4.12 ± 0.35 ^b^	0.45 ± 0.01 ^a^	0.13 ± 0.01	0.29 ± 0.11 ^a^	0.28 ± 0.01 ^a^
Hydroxycinnamic aldehydes									
coniferaldehyde	29.20	322	0.05 ± 0.00 ^a^	0.12 ± 0.00 ^b^	<LOQ ▪▪	0.19 ± 0.01 ^c^	<LOQ ▪▪	0.07 ± 0.01 ^ab^	0.06 ± 0.00 ^d^
Flavan-3-ol									
(+)-catechin	9.0	280	<LOQ ▪▪▪	0.01 ± 0.00 ^ab^	0.04 ± 0.01 ^b^	1.19 ± 0.01 ^d^	0.02 ± 0.00 ^ab^	0.03 ± 0.00 ^ab^	0.18 ± 0.01 ^c^
Flavonols									
rutin	31.9	255	25.91 ± 1.31 ^d^	5.07 ± 2.31 ^a^	2.11 ± 0.30 ^a^	6.35 ± 0.74 ^a^	<LOQ ▪▪	14.35 ± 2.19 ^c^	8.57 ± 6.03 ^b^
myricitrin	33.4	263	2.44 ± 0.71 ^ab^	0.52 ± 0.07 ^a^	5.71 ± 1.21 ^c^	2.61 ± 0.13 ^ab^	<LOQ ▪▪	2.80 ± 0.13 ^b^	<LOQ ▪▪
myricetin	34.5	360	0.05 ± 0.01 ^a^	0.56 ± 0.03 ^d^	<LOQ ▪▪	1.06 ± 0.01 ^e^	0.39 ± 0.07 ^c^	0.04 ± 0.00 ^a^	0.18 ± 0.01 ^b^
quercetin	41.0	360	1.20 ± 0.13 ^d^	0.24 ± 0.02 ^a^	0.51 ± 0.06 ^b^	0.83 ± 0.08 ^c^	0.55 ± 0.04 ^b^	0.21 ± 0.08 ^a^	0.22 ± 0.02 ^a^
kaempferol	45.3	360	0.10 ± 0.01 ^b^	0.03 ± 0.00 ^a^	<LOQ ▪▪	0.11 ± 0.00 ^b^	<LOQ ▪▪	0.07 ± 0.03	<LOQ ▪▪
Flavanone									
naringenin	43.5	280	0.17 ± 0.01 ^ab^	0.45 ± 0.07 ^bc^	<LOQ ▪▪	0.74 ± 0.14 ^c^	0.07 ± 0.02 ^a^	0.07 ± 0.03	0.02 ± 0.00

t_R_—retention time; λ_max_—maximum wavelength; LOQ—Limit of quantification. LOQ ▪ (4-Hydroxybenzoic acid) 3.13 µg/mL; LOQ ▪▪ (ellagic acid; *p*-Coumaric acid; coniferaldehyde; rutin; myricitrin; myricetin; quercetin; kaempferol; naringenin) 0.78 µg/mL; LOQ ▪▪▪ ((+)-catechin) 1.56 µg/mL; Means within the same row followed by different letters are significantly different (*p* < 0.05) according to LSD Test. Mel—*A. melanoxylon*; Lon—*A. longifolia*; Cy—*A. cyclops*; Re—*A. retinodes*; Py—*A. pycnantha*; Mea—*A. mearnsii*; De—*A. dealbata.*

## Data Availability

Not applicable.

## Supplementary Materials

The following supporting information can be downloaded at https://www.mdpi.com/article/10.3390/plants12193486/s1, Scheme S1. Detailed photographs of some Acacia green pods samples; Figure S1. Illustration of flavonoids’ basic structures combining carbon atoms and ring indices; Table S1. Chemical standards, reagents, and strains used; Figure S2. Mass spectra of flavonoids (A) MS2 of kaempferol (*m*/*z* 285.0396); and (B) MS2 of quercetin (*m*/*z* 301.0356); Table S2. Target flavonoids quercetin and kaempferol identified by LC-ESI-HRMS/MS in negative mode; Figures S3–S9. Tandem mass spectra of protonated and deprotonated.
