# Supplementary material for: First Chemical Profile Analysis of Acacia Pods
Source: Plants (Basel). 2023 Oct 5;12(19):3486. doi: 10.3390/plants12193486 (PMC10575431; doi:10.3390/plants12193486)
Supplement: Supplementary file 1 [file plants-12-03486-s001.zip › plants-2622264-supplementary.pdf]

plants

MDPI

Supplementary Materials

First Chemical Profile Analysis of Acacia Pods

Soraia I. Pedro [1,2], Tiago A. Fernandes [3,4], Ângelo Luís [5], Alexandra M. M. Antunes [3], José C. Gonçalves [1,6], Jorge Gominho [2], Eugenia Gallardo [5,7] and Ofélia Anjos [1,2,6*]

[1] Centro de Biotecnologia de Plantas da Beira Interior, Castelo Branco, Portugal; soraia_p1@hotmail.com (S.I.P.); jcgoncalves@ipcb.pt (J.C.G.); ofelia@ipcb.pt (O.A.)

[2] Centro de Estudos Florestais (CEF), Laboratório Associado TERRA, Instituto Superior de Agronomia, Universidade de Lisboa, Lisboa, Portugal; soraia_p1@hotmail.com (S.I.P.); jgominho@isa.ulisboa.pt (J.G.)

[3] Centro de Química Estrutural (CQE), Institute of Molecular Sciences, Departamento de Engenharia Química, Instituto Superior Técnico (IST), Universidade de Lisboa, Avenida Rovisco Pais, 1049-001 Lisboa, Portugal; tiago.a.fernandes@tecnico.ulisboa.pt (T.A.F.); alexandra.antunes@tecnico.ulisboa.pt (A.M.M.A.)

[4] Departamento de Ciências e Tecnologia (DCeT), Universidade Aberta, Lisboa, Portugal;

[5] Centro de Investigação em Ciências da Saúde (CICS-UBI), Universidade da Beira Interior, Covilhã, Portugal; afluis27@gmail.com (Â.L.); egallardo@fcsaude.ubi.pt (E.G.)

[6] CERNAS-IPCB, Research Centre for Natural Resources, Environment and Society, Polytechnic Institute of Castelo Branco, 6001-909 Castelo Branco, Portugal

[7] Laboratório de Fármaco-Toxicologia—UBIMedical, Universidade da Beira Interior, Covilhã, Portugal

* Correspondence: ofelia@ipcb.pt; Tel.: +351-272-339-900.

Supporting Information contains: Detailed photographs of some Acacia green pods samples used in this study (Scheme S1); Illustration of flavonoids' basic structures combining carbon atoms and ring indices (Figure S1); Chemical standards, reagents and strains used (Table S1); Mass spectra of flavonoids (A) MS^2 of kaempferol (m/z 285.0396); and (B) MS^2 of quercetin (m/z 301.0356); (Figure S2); Target flavonoids quercetin and kaempferol identified by LC-ESI-HRMS/MS in negative mode (Table S2); Tandem mass spectra of protonated and deprotonated (Figures S3-9).

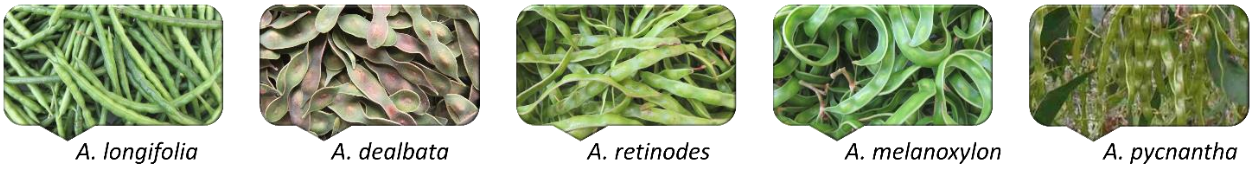

Scheme S1. Detailed photographs of some Acacia green pods samples.

Table S1. Chemical standards, reagents and strains used: their supplier and CAS number and their purity.

Analyte HPLC-DAD	Supplier	CAS Number	Purity (%)
catechol	Alfa Aesar, by Thermo Fisher Scientific	120-80-9	99.0
4-hydroxybenzoic acid	Alfa Aesar, by Thermo Fisher Scientific	99-96-7	99.0
ellagic acid	Sigma-Aldrich, St. Louis, MS, USA	476-66-4	≥ 95.0
syring acid	Supelco	530-57-4	
vanillin acid	Supelco	121-34-6	97.0
4-hydroxybenzaldehyde	Sigma-Aldrich, St. Louis, MS, USA	123-08-0	98.0
vanillin	Extrasynthese	121-33-5	99.0
syringaldehyde	Sigma-Aldrich, St. Louis, MS, USA	134-96-3	98..0
chlorogenic acid	Extrasynthese	327-97-9	99.0
caffeic acid	Extrasynthese	331-39-5	99.0
p-coumaric acid	Sigma-Aldrich, St. Louis, MS, USA	501-98-4	98.0
trans-cinnamic acid	Merck	140-10-3	97.0
4-hydroxy-3-methoxycinnamaldehyde	Supelco	458-36-6	98.0
furfural	Sigma-Aldrich, St. Louis, MS, USA	98-01-1	99.0
5-methylfurfural	Sigma-Aldrich, St. Louis, MS, USA	620-02-0	99.0
(+)-catechin	TCL Europe N.V., Zwijndrecht, Begium	154-23-4	97.0
(-)-epicatechin	TCL Europe N.V., Zwijndrecht, Belgium	490-46-0	97.0
rutin	Sigma-Aldrich, St. Louis, MS, USA	153-18-4	99.0
myricitrin	Sigma-Aldrich, St. Louis, MS, USA	17912-87-7	99.0
myricetin	Extrasynthese	529-44-2	≥96.0
quercetin	Supelco	117-39-5	≥95.0
kaempferol	Extrasynthese	520-18-3	≥97.0
4',5,7-trihydroxyflavanone	Alfa Aesar, by Thermo Fisher Scientific	67604-48-2	97.0
**Antioxidant Activity**			
folin ciocalteu	Sigma-Aldrich, St. Louis, MS, USA	12111-13-6	
potassium carbonate	Alfa Aesar, by Thermo Fisher Scientific	584-08-7	99.0
aluminum chloride	Alfa Aesar, by Thermo Fisher Scientific	7446-70-0	99.0
potassium acetate	Carlo Erba	127-08-2	99.0
DPPH	Sigma–Aldrich, St. Louis, MS, USA	1898-66-4	
methanol	Fluka,Milwaukee, WI, USA	67-56-1	99.0
β-carotene	Sigma-Aldrich, St. Louis, MS, USA	7235-40-7	>93.0
linoleic acid	TCI Europe N.V., Zwijndrecht, Belgium	60-33-3	99.0
tween 40	Riedel-de Häen, Seelze, Germany	9005-66-7	
chloroform	Scharlab, Barcelona, Spain	67-66-3	99.0
BHT - butylated hydroxytoluene	Fluka, Milwaukee, WI, USA	128-37-0	99.0
**Antimirobial Activity**			
Reference strains	American Type Culture Collection (ATCC, Manassas, VT, USA) or BCCM/LMG Bacteria Collection Belgian Co-Ordinated Collections of Microorganisms, Gent, Belgium		
glycerol	Himedia, Mumbai, India	56-81-5	99.0
infusion agar (BHI)	Liofilchem, Roseto degli Abruzzi, Italy		
müeller-Hinton agar (MHA)	Liofilchem, Roseto degli Abruzzi, Italy		
sabouraud Dextrose Agar (SDA)	Liofilchem, Roseto degli Abruzzi, Italy		
dimethyl sulfoxide (DMSO)	Sigma–Aldrich, St. Louis, MS, USA	67-68-5	
resazurin	TCI Europe N.V., Zwijndrecht, Belgium	62758-13-8	
müeller-Hinton broth (MHB)	Liofilchem, Roseto degli Abruzzi, Italy		
**LC-ESI-HRMS/MS Analysis**			
acetonitrile	Sigma-Aldrich, St. Louis, MS, USA	75-05-8	99.0
ethanol	Sigma-Aldrich, St. Louis, MS, USA	64-17-5	99.0
formic acid	Sigma-Aldrich, St. Louis, MS, USA	64-18-6	>95.0

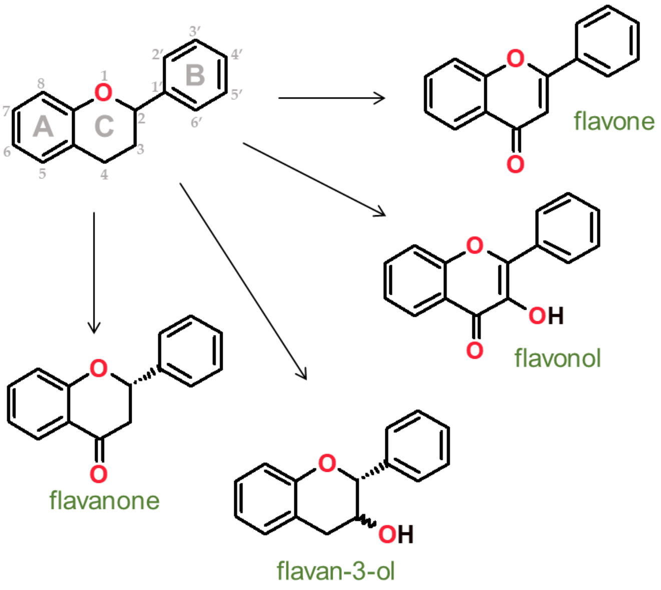

Figure S1. Illustration of flavonoids' basic structures combining carbon atoms and ring indices.

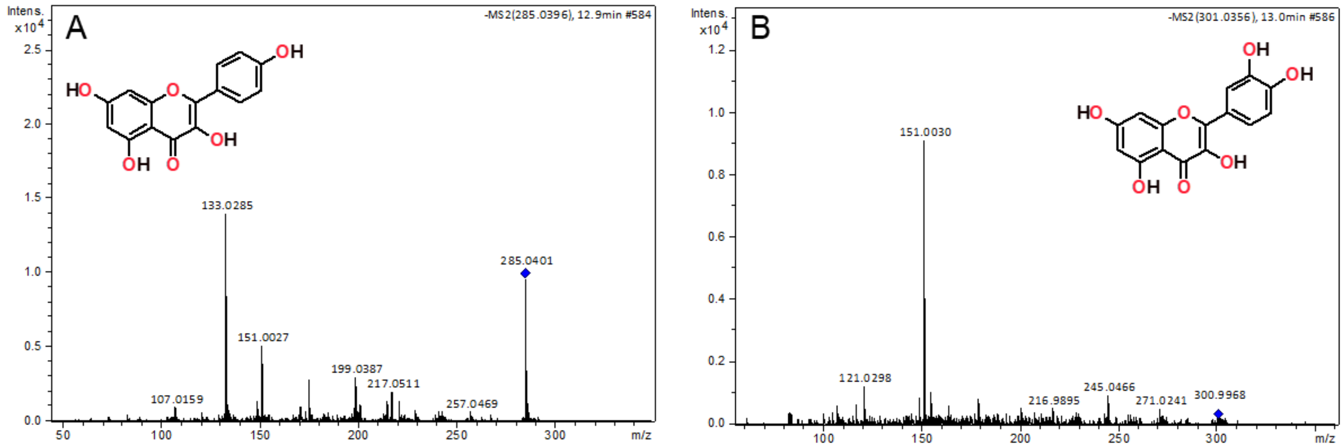

Figure S2. Mass spectra of flavonoids (A) MS² of kaempferol (m/z 285.0396); and (B) MS² of quercetin (m/z 301.0356).

Table S2. Target flavonoids quercetin and kaempferol identified by LC-ESI-HRMS/MS in negative mode.

Identified Compound	RT[a] (min)	Molecular Formula	$[M-H]^-$ (*m/z* exp)	error (ppm)	Fragment ions *m/z* (error ppm, molecular formula)
**myricetin**	12.0	$C_{15}H_{10}O_8$	317.0300	-0.9	271.0239 (-3.4, $C_{14}H_7O_6^-$)
					178.9983 (-1.7, $C_8H_3O_5^-$)
					151.0034 (-1.9, $C_7H_3O_4^-$)
					137.0239 (-3.8, $C_7H_5O_3^-$)
**quercetin**	13.0	$C_{15}H_{10}O_7$	301.0356	0.7	245.0452 (1.4, $C_{13}H_9O_5^-$)
					178.9985 (-0.5, $C_8H_3O_5^-$)
					151.0030 (-4.5, $C_7H_3O_4^-$)
**kaempferol**	13.2	$C_{15}H_{10}O_6$	285.0396	-3.0	215.0358 (3.8, $C_{12}H_7O_4^-$)
					199.0385 (-7.9, $C_{12}H_7O_3^-$)
					170.9620 ($C_{12}H_7O_4^-$)
					149.0237 (-4.8, $C_8H_5O_3^-$)
					133.0293 (-1.5, $C_8H_5O_2^-$)

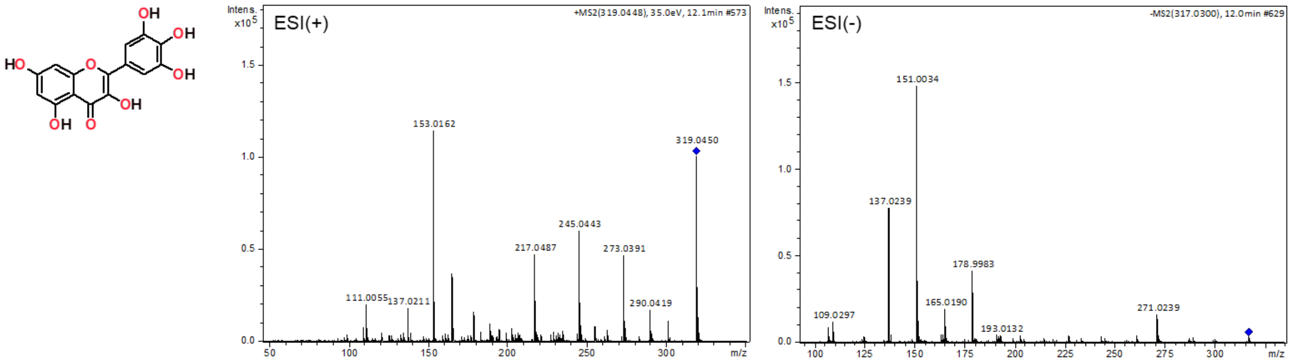

ESI(+)

Figure S3. Tandem mass spectrum of the protonated and deprotonated molecule of myricetin.

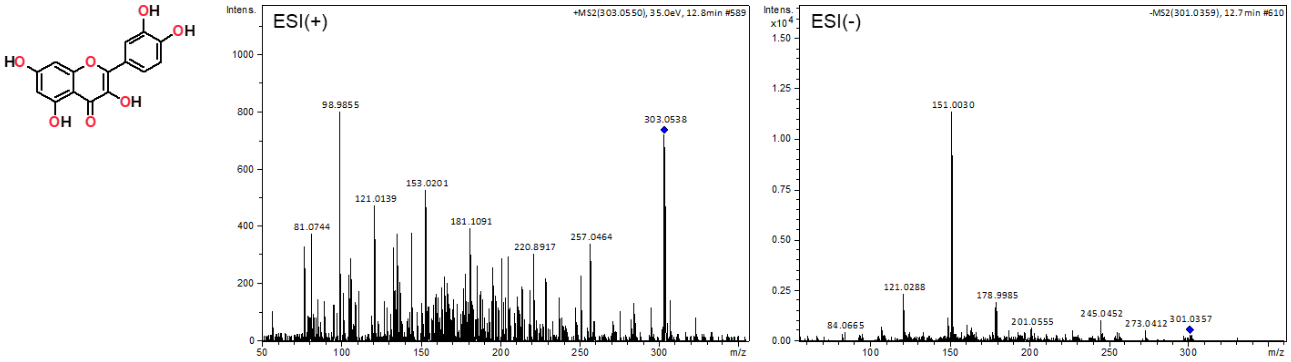

Figure S4. Tandem mass spectrum of the protonated and deprotonated molecule of quercetin.

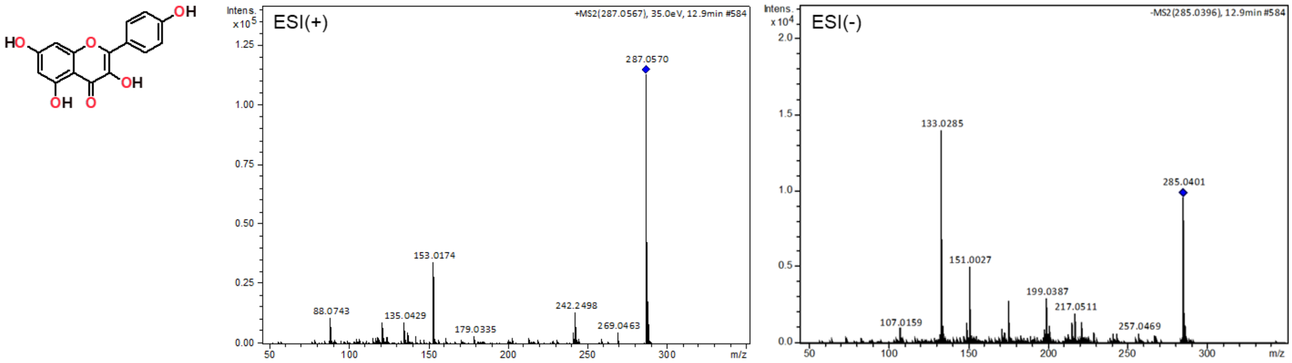

Figure S5. Tandem mass spectrum of the protonated and deprotonated molecule of kaempferol.

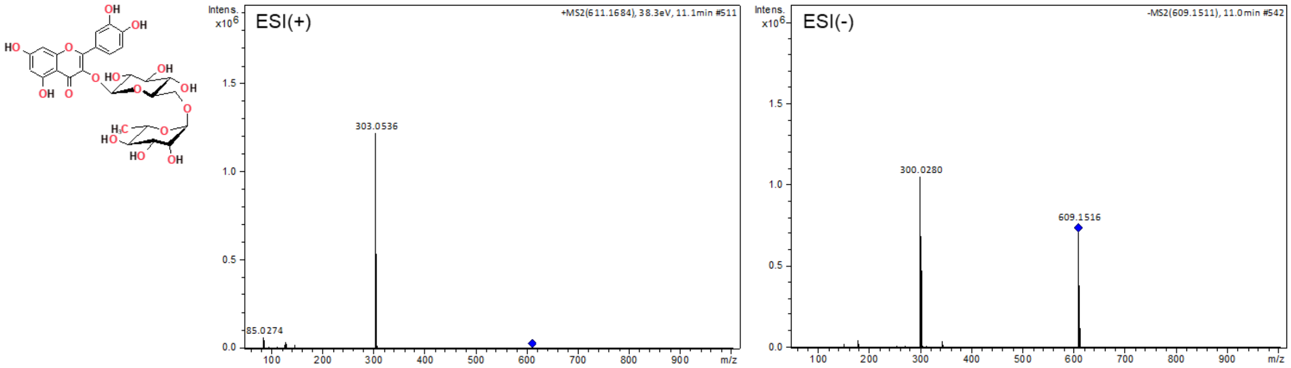

Figure S6. Tandem mass spectrum of the protonated and deprotonated molecule of rutin.

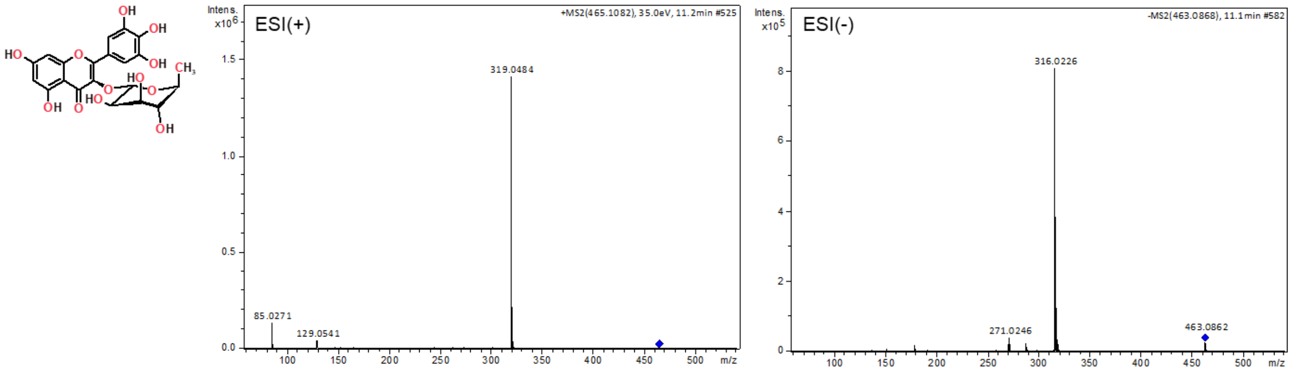

Figure S7. Tandem mass spectrum of the protonated and deprotonated molecule of myricitrin.

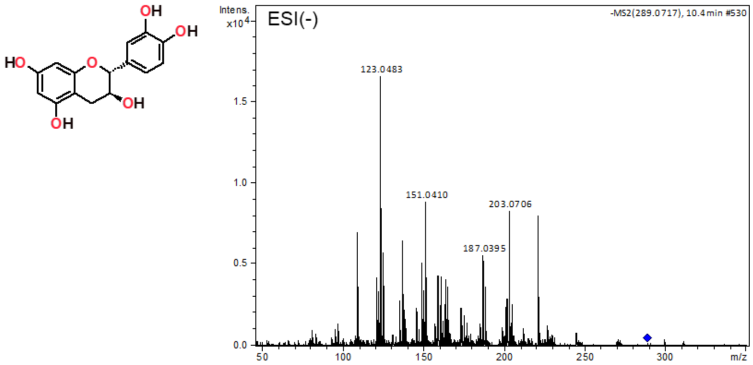

Figure S8. Tandem mass spectrum of the deprotonated molecule of (+)-catechin.

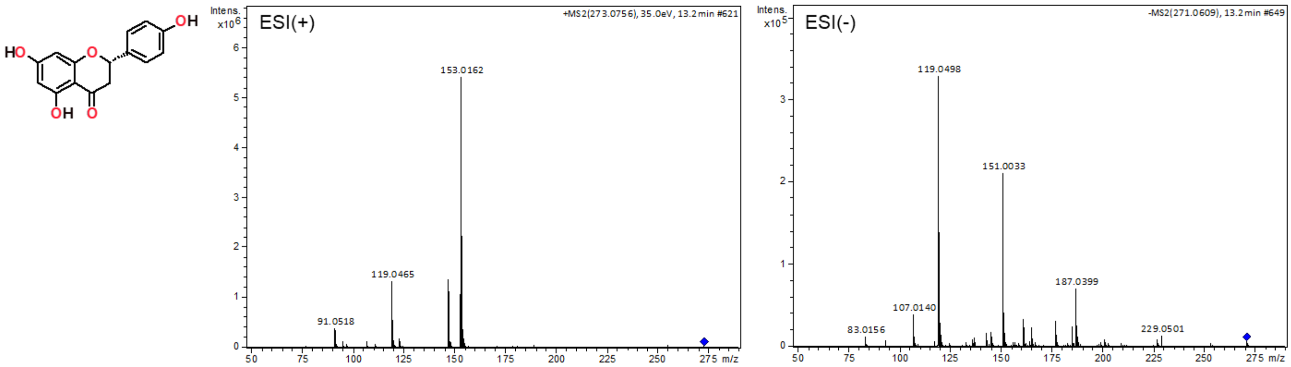

Figure S9. Tandem mass spectrum of the protonated and deprotonated molecule of naringenin.
